# Social support perceived by breastfeeding mothers and its impacts on breastfeeding

**DOI:** 10.1590/0034-7167-2025-0125

**Published:** 2026-07-17

**Authors:** Luciane Cristina Rodrigues Fernandes, Julia Melo Campanha, Clara Fróes de Oliveira Sanfelice, Reginaldo Roque Mafetoni, Elenice Valentim Carmona

**Affiliations:** I Universidade Estadual de Campinas. Campinas, São Paulo, Brazil

**Keywords:** Perceived Social Support, Social Support, Breastfeeding Women, Breast Feeding, Maternal-Child Nursing., Apoyo Social Percibido, Apoyo Social, Madres Lactantes, Lactancia, Enfermería Maternoinfantil.

## Abstract

**Objectives:**

to understand the support network of breastfeeding mothers with children born in two teaching hospitals, its effectiveness in the postpartum period, and its impact on breastfeeding.

**Methods:**

a prospective cohort study was conducted in two public hospitals accredited to the Baby-Friendly Hospital Initiative. For analysis, the Statistical Package for the Social Sciences software was used.

**Results:**

of 304 postpartum women, 295 (97.04%) mentioned support from the hospital’s health team, and 283 (93.09%) reported having a support network in the postpartum period. Support network became effective in the first month postpartum (p=0.0001), favoring daytime rest (p=0.0005) and having a positive relationship with exclusive breastfeeding in the sixth month (p=0.0424).

**Conclusions:**

most women considered the healthcare team as a source of support for breastfeeding and mentioned that they would have a support network after discharge, which materialized with a positive outcome for breastfeeding.

## RESUMEN

**Objetivos:**

comprender la red de apoyo de las madres lactantes con hijos nacidos en dos hospitales universitarios, su efectividad en el período posparto y su impacto en la lactancia materna.

**Métodos:**

Estudio de cohorte prospectivo realizado en dos hospitales públicos acreditados por la Iniciativa Hospital Amigo del Niño. Para el análisis se utilizó el *software Statistical Package for the Social Sciences*.

**Resultados:**

de 304 mujeres en el posparto, 295 (97,04%) mencionaron haber recibido apoyo del equipo de salud del hospital y 283 (93,09%) indicaron contar con una red de apoyo durante el período posparto. La red de apoyo resultó efectiva durante el primer mes posparto (p=0,0001), favoreciendo el descanso diurno (p=0,0005) y mostrando una relación positiva con la lactancia materna exclusiva al sexto mes (p=0,0424).

**Conclusiones:**

la mayoría de las mujeres consideraron al equipo de atención médica como una fuente de apoyo para la lactancia materna y mencionaron que contarían con una red de apoyo tras el alta, lo cual se tradujo en un resultado positivo para la lactancia.

## INTRODUCTION

The World Health Organization (WHO) recommends that all children receive exclusive breastfeeding (EBF) for the first 6 months of life. From this period, breastfeeding (BF) should continue until the age of two, along with the introduction of complementary foods^(1)^. Human milk is a complete food that protects newborns (NBs) against infections, reducing the possibility of developing comorbidities such as obesity and overweight. It is related to the maturation of the Central Nervous System, cognitive development, better performance on intelligence tests, and higher income in adulthood^(1-3)^. In addition to promoting child growth and development, it benefits BF mothers by reducing the risk of ovarian and breast cancer, lowering the risk of type II diabetes mellitus, and allowing for longer intervals between pregnancies, since EBF acts as a natural contraceptive method under certain conditions^(1,2)^.

In Brazil and worldwide, promoting BF has the potential to reduce infant morbidity and mortality, since approximately 16% of annual infant deaths are associated with inadequate BF^(2,4)^. Furthermore, nearly 1 million cases of childhood obesity can be attributed to insufficient BF^(4)^. In 2012, the WHO set a global goal for the rate of EBF in the first 6 months of life to reach 50% by 2025^(5)^. In Brazil, the latest population-based household survey conducted by the 2019 Brazilian National Study of Infant Feeding and Nutrition showed that the prevalence of EBF in children under 6 months is below WHO recommendations, standing at 45.8%^(6)^. Another national study indicated that, despite the increase in EBF rates between 2015 and 2019, mixed BF rates also grew, especially in regions with low levels of adequate nutrition^(7)^.

Furthermore, in the current context, women play diverse roles in society, and reconciling these roles with motherhood is challenging, making the daily experience of BF a complex one that demands the involvement of healthcare professionals, family, community, and institutions. A woman’s adaptation to the role of a nursing mother, the one who breastfeeds, is not spontaneous or natural, as it is influenced by sociocultural norms and preconceived personal expectations^(8)^. Therefore, this is a complex process that requires a redefinition of women’s social identity and adaptation of their routine to infants’ needs^(8)^. These changes evoke psychological and social consequences that may go unnoticed by those who should be supporting women, but which can represent challenges and barriers to establishing BF.

The burden of motherhood on women who raise children alone has significant psychological and physical repercussions^(9)^. Feelings of exhaustion and vulnerability after childbirth, recovery from a cesarean section, isolation, and a lack of knowledge about how to care for a baby undermine women’s self-confidence^(9,10)^. Furthermore, the emergence of factors such as the perception of failure in BF, feelings of guilt, lack of social support, and insufficient maternity leave time poses a challenge to BF continuity^(9,10)^. In these cases, the absence of support is one of the factors that contribute to early weaning, while social support contributes significantly to the establishment of EBF^(9-11)^.

Thus, perceived social support, or support network (SN), is one of the fundamental aspects for successful BF, and can be defined in various ways in the literature. It is a system made up of different individuals belonging to the social sphere who provide support in different areas of life: emotional, material, educational, among others^(12)^. A study conducted in the United Kingdom identified three types of SNs: “extensive support”, for mothers who received support from family, friends, and professionals; “family support”, for those who were primarily supported by family, excluding professionals; and “low support”, for those who reported the least support among all potential supporters^(13)^. And women who breastfed for more than two months were more likely to report widespread support^(13)^.

BF is a phase of the pregnancy-postpartum cycle that should not be experienced in isolation by BF mothers, because, with the support of SNs, there is a relief of the burden on postpartum women, which allows this period to be experienced in a healthier way and ensures BF continuity^(14-16)^. Thus, the objective is to understand SNs of BF women and their impacts on BF. It is understood that this knowledge can facilitate the development of strategies to promote and protect BF.

## OBJECTIVES

To understand the SN of BF mothers with children born in two teaching hospitals in the countryside of São Paulo, its effectiveness in the postpartum period, and its impacts on BF.

## METHODS

### Ethical aspects

The study complied with all recommendations of Resolution 466 of December 12, 2012 of the Brazilian National Health Council, and was approved by the *Universidade Estadual de Campinas* (UNICAMP) Research Ethics Committee. All participants signed the Informed Consent Form and were informed that they could withdraw their consent at any time, even after signing, without suffering any kind of burden or embarrassment.

### Study design, location, and participants

This is a prospective cohort study conducted in two public teaching hospitals accredited to the Baby-Friendly Hospital Initiative, which provide care to pregnant women throughout their pregnancy and postpartum period, serving as regional referral centers for pregnancies with usual, medium, or high-risk pregnancies during labor and childbirth. These hospitals will be referred to here as hospital A and hospital B. The sample size was 304 postpartum women. This sample was divided proportionally between hospitals A and B: 156 and 148 participants, respectively.

This was a convenience sample and, as such, consisted exclusively of cisgender women. Therefore, throughout the article, we will use the terms “postpartum women”, “women”, and “maternal”.

### Inclusion criteria

The study included women with BF children who did not require observation in a neonatal intensive or intermediate care unit at any time, aged 18 years or older, with or without comorbidities, provided that such comorbidities did not prevent them from BF, more than 24 hours postpartum, BF a child from a single pregnancy, and fluent in Brazilian Portuguese.

### Exclusion criteria

Women who presented limitations in communication, as well as difficulty in understanding the questions, according to medical diagnosis and/or the researcher’s perception, were excluded. Women who were separated from their children while at home or in cases where one of the members of the dyad (mother or infant) died were also discontinued.

### Collection procedure

Data were collected from medical records, and interviews were conducted using a structured questionnaire developed for this study. The questionnaire included data for sample characterization, obstetric and BF data, information about the birth, as well as an investigation into postpartum women’s recognition of SN.

Data collection occurred in four distinct timeframes. In the first timeframe (T0), data were collected from postpartum women in rooming-in, 24 hours or more after childbirth; in the second, third, and fourth timeframes, data were collected from these same women by telephone one month (T1), three months (T2), and six months (T3) after the baby’s birth. As a criterion for continuing the interviews, it was necessary that the woman was BF or that the baby was receiving milk from their nursing mothers in some way. Thus, in T1, 272 postpartum women were interviewed (90.13% of the initial sample), in T2, 224 (91.80% of T1), and in T3, we had 191 (96.46% of T2) interviews.

Data collection in rooming-in took place from September 2022 to May 2024, concluding contacts in T3 in November 2024.

### Data analysis

A descriptive and inferential statistical analysis was performed using the Statistical Package for the Social Sciences software. For the distribution of sociodemographic and clinical variables, descriptive analysis was performed by calculating frequencies and percentages, as well as measures of central tendency and dispersion. Associations among groups were assessed using the chi-square test. When the chi-square test assumptions were not met, McNemar’s test was applied. The significance level adopted in the analyses was 5%.

## RESULTS

A total of 304 women were interviewed, 156 (51.32%) from hospital A and 148 (49.68%) from hospital B, as predicted in the sample size calculation. Table 1 presents the sociodemographic profile of interviewees.

**Table 1:** Sociodemographic profile of interviewees in rooming-in (N=304), Campinas, São Paulo, Brazil, 2023-2024

	n	%
**Age range**		
18 to 34 years old	232	76.32
35 to 46 years old	72	23.68
**Skin color (self-reported)**		
Brown	155	50.99
White	117	38.49
Black	31	10.20
Yellow	1	0.33
**Marital status**		
United	265	87.17
Not united	39	12.83
**Study time (years)**		
< five	9	2.96
Six to nine	42	13.81
Ten to 13	227	74.67
> 14	26	8.55
**Family income**		
No income	2	0.66
Less than one minimum wage	26	8.55
One to two minimum wages	112	36.84
Three to five minimum wages	115	37.83
More than five minimum wages	34	11.18
Do not know/did not want to say	15	4.93

The interviewees’ mean age was 29.30 years (median: 29; min.: 18; max.: 46; SD: 6.53). The mean length of education was 11.40 years (min.: 2; max.: 22; SD: 2.37). In accordance with the guidelines of the Brazilian Health System (In Portuguese, *Sistema Único de Saúde* - SUS), the prevalent municipalities of residence were those where the hospitals are located, followed by neighboring municipalities. Only nine (3%) women resided in rural areas.

Slightly more than half (n=164; 53.95%) of respondents stated that they were employed, and among them, 148 (80%) were entitled to maternity leave. Regarding marital status, 265 (87.17%) of women declared that they were in a relationship (being married, in a stable union, or living in the same residence as their partner), with a mean length of relationship of 6.54 years (min.: 3 months; max.: 27.16 years; SD: 5.51 years).

Concerning gestational history, the mean number of pregnancies was 2.51 (min.: 1; max.: 9; SD: 1.52), and 106 (34.87%) were primiparous. Only one woman (0.3%) did not have prenatal care. However, 22 (7.26%) had fewer than six prenatal visits, falling short of the Ministry of Health recommendation. The mean number of prenatal visits was 10.53 (min.: 2; max.: 23; SD: 3.48). As for the mode of childbirth, 157 (51.64%) had their children via surgical childbirth.

Regarding fetal maturity, 272 (89.47%) NBs presented a Capurro method greater than or equal to 37 weeks (mean: 38.66; min.: 35; max.: 41.43 weeks; SD: 1.35). The mean birth weight was 3,122.77g (min.: 2,004 g; max.: 4,310 g; SD: 468.58 g).

Concerning BF history, 239 (78.62%) postpartum women stated that they had been breastfed. There were 194 (63.82%) women who already had other children, and 185 (95.36%) had some degree of experience with BF.

When asked if the interviewees felt supported by the hospital staff to breastfeed, 295 (97.04%) said yes: the professional category was not asked here, but whether they had received support in general. Among those who acknowledged the support, 275 (90.46%) reported receiving it whenever they needed it, and 20 (6.58%) mentioned that it occurred a few times throughout the day, not representing the totality of their needs. Only nine (3%) did not feel supported by the staff. Although most felt supported to breastfeed, 278 (90.46%) reported not feeling confident to breastfeed with the information they received at the hospital.

In relation to the use of infant formula, supplementation was prescribed for 76 (25%) of NBs. However, 87 (28.62%) postpartum women stated that their child had received milk other than their own. Regarding mothers’ desire to continue BF after hospital discharge, only one woman stated that she would not continue BF, citing the need to work as the reason for interrupting lactation.

As for the presence of a companion, 280 (92.11%) women had one during labor and childbirth. Only 77 (25.3%) breastfed in the delivery room, and 120 (39.47%) reported some type of difficulty BF in the first 24 hours of the baby’s life. Having a companion during labor and childbirth did not impact the possibility of BF in the delivery room (p=0.9692), the offer of formula during hospitalization (p=0.9764), or the mother’s perception of BF difficulties (p=0.8397). There was no statistical significance in the presence of a companion during the parturition process for in-hospital BF, as shown in Table 2.

**Table 2:** Presence of a companion during labor and childbirth and its relationship with in-hospital breastfeeding (N=304), Campinas, São Paulo, Brazil, 2023-2024

Variable	Companion during labor and childbirth				*p* value^*^
	No		Yes		
	n	%	n	%	
**Breastfeeding in the delivery room**					0.9692^*^
No	18	75.00	209	74.64	
Yes	6	25.00	71	25.36	
**Offering infant formula during hospitalization**					0.9764^*^
No	17	70.83	197	71.12	
Yes	7	29.17	80	28.88	
**Difficulty breastfeeding**					0.8367^*^
No	15	62.50	169	60.36	
Yes	9	37.50	111	39.64	

* *p value obtained using the chi-square test.*

When asked about SNs that could help them during the postpartum period, 283 (93.09%) said they could count on help after discharge; ten (3.29%) were unsure if they would have someone who could assist them; and 11 (3.62%) said they had no SN. The partner (husband, boyfriend, baby’s father) was the most frequently mentioned (n=177; 62.99%) within the SN, followed by the mother (n=132; 46.98%), the mother-in-law (n=40; 14.23%), and siblings (n=32; 11.39%). Two (0.71%) women did not name who their SN would be.

As mentioned in the method, after the first interview in the rooming-in setting, a follow-up telephone interview was conducted one month postpartum (T1). For dyads that continued BF, further contacts were made three (T2) and six (T3) months after the birth. Sample size varied throughout the interviews, from the first telephone contact one month after the baby’s birth (T1). A sample of 272 postpartum women was selected (90.13% of the initial sample); of these, 30 women (11.02%) were no longer BF their babies. At T2, 224 postpartum women (92.56% of the previous sample) were interviewed, with 26 (11.61%) cases of weaning, and at T3, 191 postpartum women (96.46% of T2) were interviewed, with 19 (9.95%) cases of weaning. Throughout the data collection, only two (0.66%) postpartum women were discontinued. It was not possible to contact 55 (18.09%) women.

In T1, women were asked if they had experienced SN during the first month postpartum, and prior recognition of SN was statistically significant in relation to its occurrence postpartum (Table 3).

**Table 3:** Effectiveness of the home support network questioned in T1, Campinas, São Paulo, Brazil, 2023-2024

Belief that there will be SN at home (Questioned in T0)	Presence of SN in the first month postpartum		*p* value^*^
	No	Yes	
No	4	6	**< 0.0001**
Yes	33	219	

* *p value obtained using McNemar’s test; SN - support network; T0 - time the interview was conducted: rooming-in.*

When assessing the impact of SN on pacifier use in the baby’s first month of life, there was no statistical significance (p=0.6604), as well as formula introduction throughout the baby’s 6 months of life, questioned at T1 (p=0.3025), T2 (p=0.3746), and T3 (p=0.0709).

However, a positive relationship was found between women who had SN and the possibility of rest throughout the day, showing that it favored EBF at six months of age (p=0.0424). Having SN did not interfere with nighttime rest (Table 4). Among the women who stated that they were exclusively BF their babies at six months of age (n=76), 15 of them (19.74%) offered formula at some point in the previous months. Thus, among all the interviewees, only 20.07% (n=61) had EBF until six months, and for this, SN showed statistical significance, as shown in Table 4.

**Table 4:** Support network in the first month postpartum and its association with breastfeeding, Campinas, São Paulo, Brazil, 2023-2024

Variable	SN in the first month postpartum				*p* value^*^
	No		Yes		
	n	%	n	%	
**Rest throughout the day (T1)**					**0.0005**
No	22	56.41	66	28.33	
Yes	17	43.59	167	71.67	
**Rest at night (T1)**					0.5510
No	16	41.03	84	36.05	
Yes	23	58.97	149	63.95	
**Feeding a baby at 6 months**					**0.0424**
Others^#^	19	79.17	96	57.49	
EBF	5	20.83	71	42.51	
**Exclusive breastfeeding until six months**					**0.0490**
No	35	89.74	176	75.54	
Yes	4	10.26	57	24.46	

* *p value obtained using the chi-square test; SN - support network; EBF - exclusive breastfeeding; T1 - one month postpartum; #Other: partial, mixed or supplemental breastfeeding.*

## DISCUSSION

The sociodemographic data are similar to the findings of research carried out by the Stork Network and Healthy Birth^(14)^ in public and SUS-affiliated hospitals across Brazil, which show a higher percentage of women under 35 years of age and of mixed race. However, there was a discrepancy between the data regarding the length of education found in the public sector, since, in the Stork Network research, it was ten years of study. In this sample, 100% assisted by the SUS, it was similar to the education found in the private sector, which was mostly 11 to 13 years^(14)^.

The majority of women interviewed (97.04%) felt supported by the hospital staff, a protective factor in overcoming BF difficulties. This is supported by a study that revealed that women who perceived adequate BF support while still in the hospital were less likely to experience BF difficulties compared to those who considered this support inadequate^(15)^.

Despite feeling supported by the team, they reported feeling insecure about BF based on the information received, highlighting the importance of strengthening health education and post-discharge follow-up. The literature describes that a lack of confidence in acquired knowledge about BF may indicate insufficient educational support. This need must be met, as women who have sufficient knowledge about BF are more likely to breastfeed for longer^(16)^.

A quasi-experimental study conducted with postpartum women in the immediate postpartum period demonstrated how planned, long-term support offered by nurses is related to higher scores on the BF self-efficacy scale, which measures how much a woman believes in their ability to breastfeed. In this study, nurses’ support, through information sharing and effective communication, significantly contributed to increased maternal self-efficacy, a factor that influences the BF’s success and continuity^(17)^.

In the present study, the majority of participants (93.09%) stated that they could count on SN after discharge. The importance of family presence even before the baby’s birth is well known, especially the presence of the woman’s companion throughout the entire labor process^(18)^. The partner’s identification as the primary source of support should be seen as relevant to BF continuity, since the father’s support for more than 24 months is considered a protective factor for BF^(16)^.

SN in the rooming-in setting was confirmed in the first month postpartum, being statistically significant (p=0.0001). In other words, 92.64% of the women interviewed, after one month postpartum, validated the SN participation. By encouraging the partners of BF mothers to recognize the crucial role they play in their children’s nutrition from the beginning of BF, their active participation in the child’s daily care and other activities is stimulated, which can contribute to a more positive BF experience for mothers^(19)^. Healthcare professionals can play a role in recognizing and strengthening this role, starting from prenatal care.

In addition to the emotional and social aspects, it is known that BF can be a period of great physiological demand for mothers, since they must provide the necessary nutrients for children through their milk, which implies that they require physical and emotional care, as well as rest. The sleep/wake cycle can be drastically altered by BF, as can the secretion of hormones and neurotransmitters involved in the “fight or flight” response^(20)^. Studies suggest that increased secretion of prolactin and oxytocin reduces the maternal stress response, anxiety, and irritability^(21)^. In addition to hormonal effects, SN may be another factor in reducing the stress response. The present study showed that SN favors the possibility of rest during the daytime (p=0.0005). However, it did not show an impact on rest during the nighttime (p=0.5510), which may be due to lower availability of SN during this period.

The correlation between pacifier use and the presence of allergic rhinitis was not statistically significant (p=0.6604). A possible explanation could be a lack of knowledge about recommendations regarding pacifier use, as analyzed in a recent study that investigated parents’ knowledge and attitudes towards BF^(22)^. In this study, parents demonstrated knowledge regarding the benefits of BF for the dyad, skin-to-skin contact, rooming-in, BF on demand, and the importance of their involvement in feeding the baby; however, they showed a lack of knowledge about the impacts of pacifier use^(22)^.

Although studies indicate that the presence of a companion in the delivery room and SN in the postpartum period contribute to better BF rates^(23)^, SN did not impact formula introduction before or after discharge. There was also no statistical significance between having a companion during labor and childbirth, and BF in the delivery room, offering of in-hospital formula, as well as difficulty BF. There are some barriers to BF continuity, such as the existence of a strong generalized narrative that women’s bodies are inadequate and that they cannot produce sufficient volume and/or quality milk. It is also conveyed that formula feeding is more convenient and socially acceptable, which, obviously, is supported by formula companies to sell more of their products^(4)^.

Having overcome the difficulties encountered in the first months of BF, this study showed a positive relationship between the presence of SN, the withdrawal of formula, and the resumption of EBF up to six months, and EBF from birth to six months. This finding corroborates a study that states that the perception of a higher level of social support is related to a longer BF duration, i.e., women who perceive that they have greater support are more likely to plan and carry out prolonged BF^(20)^. Evidence suggests that social support improves BF outcomes^(13)^.

EBF continuity after hospital discharge is related to the challenges faced by BF mothers. These difficulties may include the perception of insufficient milk production, limitations in finding good BF positions, difficulties with the baby’s latch and sucking, formula supplementation introduction, inadequate knowledge about how to initiate or wean from supplementation, as well as discomfort and pain during BF^(8,24)^. Furthermore, insufficient support in coping with difficulties can be a contributing factor to early weaning. The overload generated by the need to reconcile the role of nursing mother with other daily activities leads to the inability to rest reported by postpartum women^(8,9)^. Therefore, SN proves fundamental in addressing these difficulties, caring for and strengthening BF mothers, as well as protecting and promoting BF.

Based on the literature, public health services and interventions aimed at improving BF rates should primarily target informational, social, and emotional support for mothers, reflecting an individualized approach to behavior change^(13)^. Strengthening these bonds from the prenatal period onwards, helping pregnant women recognize their SNs and identifying cases of insufficient support are crucial for developing an individualized care plan, filling the gaps that become apparent during consultations. Finally, with structured SN, the BF mother will be more likely to breastfeed, receiving EBF and prolonged BF support, protection, and promotion.

### Study limitations

The limitation of this study lies in the impossibility of describing which actions women recognize as supportive throughout the postpartum period that, from their perspective, most directly impacted the relationships surrounding BF. Therefore, the design of this study did not favor the analysis of the actions experienced by women over time that represent the support and protection they expect during BF. A qualitative study could answer questions related to the meaning of SN for postpartum women.

### Contributions to nursing and public health

This study is expected to highlight the relevance of SN to BF, providing support for political and healthcare actions, as well as future scientific research on this topic, and consequently promoting and supporting BF. The positive impacts of SN should be discussed and promoted in teaching, research, and clinical practice, as it is a low-tech care method that can be implemented in women’s daily care during the pregnancy and postpartum period.

## CONCLUSIONS

Most postpartum women recognize the hospital healthcare team as a SN. They consider that, after discharge, their partner (husband, boyfriend, baby’s father) will be a likely source of support, followed by support from their mother, mother-in-law, and siblings. One month postpartum, when women were interviewed, it was found that family SN had been established, with the women validating this experience. The presence of SN facilitated rest during the day and increased the likelihood of EBF at six months.

Thus, the recognition of SN by BF mothers can be a facilitating factor in overcoming challenges, promoting BF, and increasing the chances of prolonged BF. In this context, the healthcare team should act intentionally and proactively, starting from prenatal care, including the family, as well as assisting women in identifying and strengthening their SN, in order to help their establish and continue BF.

## Data Availability

The research data are available in a repository: https://doi.org/10.25824/redu/IJFTL1.
