# Supplementary material for: Social support perceived by breastfeeding mothers and its impacts on breastfeeding
Source: Rev Bras Enferm. 2026 Jul 17;79:e20250125. doi: 10.1590/0034-7167-2025-0125 (PMC13379194; doi:10.1590/0034-7167-2025-0125)
Supplement: Translation

# Translation: Apoio social percebido por nutrizes e seus impactos no aleitamento

**DOI:** 10.1590/0034-7167-2025-0125pt

**Authors:** Luciane Cristina Rodrigues Fernandes, Julia Melo Campanha, Clara Fróes de Oliveira Sanfelice, Reginaldo Roque Mafetoni, Elenice Valentim Carmona

**Affiliations:** I Universidade Estadual de Campinas. Campinas, São Paulo, Brasil

**Keywords:** Suporte Social Percebido, Apoio Social, Nutrizes, Aleitamento, Enfermagem Materno-Infantil.

## Abstract

**Objetivos:**

conhecer a rede de apoio de nutrizes com filhos nascidos em dois hospitais de ensino, sua efetivação no período puerperal e seus impactos no aleitamento.

**Métodos:**

estudo de coorte prospectivo, realizado em dois hospitais públicos credenciados à Iniciativa Hospital Amigo da Criança. Para análise, foi usado o *software Statistical Package for the Social Sciences*.

**Resultados:**

de 304 puérperas, 295 (97,04%) mencionaram apoio da equipe de saúde do hospital, e 283 (93,09%) referiram ter rede de apoio no pós-parto. A rede de apoio se efetivou no primeiro mês pós-parto (p=0,0001), favorecendo o descanso diurno (p=0,0005) e tendo relação positiva com aleitamento exclusivo no sexto mês (p=0,0424).

**Conclusões:**

a maioria das mulheres considerou a equipe de saúde como fonte de apoio à amamentação e mencionou que teria rede de apoio após a alta, a qual se efetivou com desfecho positivo para o aleitamento.

## INTRODUÇÃO

A Organização Mundial da Saúde (OMS) recomenda que toda criança receba aleitamento materno exclusivo (AME) nos primeiros 6 meses de vida. A partir deste período, o aleitamento materno (AM) deve continuar até os dois anos, estando associado à introdução de alimentos complementares^(1)^. O leite humano é um alimento completo, que protege o recém-nascido (RN) contra infecções, reduzindo a possibilidade de desenvolvimento de comorbidades como obesidade e sobrepeso. Está relacionado à maturação do Sistema Nervoso Central, desenvolvimento cognitivo, melhor desempenho em testes de inteligência e maior renda na vida adulta^(1-3)^. Além de favorecer o crescimento e desenvolvimento infantil, traz benefícios às nutrizes, ao diminuir o risco de câncer de ovário e de mama, menor risco de diabetes mellitus tipo II, permitindo um maior intervalo entre as gestações, uma vez que o AME atua como um método contraceptivo natural, sob determinadas condições^(1,2)^.

No Brasil e no mundo, a promoção da amamentação tem potencial para reduzir a morbimortalidade infantil, uma vez que cerca de 16% das mortes infantis anuais estão associadas à amamentação inadequada^(2,4)^. Além disso, quase 1 milhão de casos de obesidade infantil podem ser atribuídos à amamentação insuficiente^(4)^. Em 2012, a OMS estabeleceu como meta global que, até 2025, a taxa de AME nos primeiros 6 meses de vida alcance 50%^(5)^. No Brasil, o último inquérito populacional domiciliar conduzido pelo Estudo Nacional de Alimentação e Nutrição Infantil de 2019 apresentou que a prevalência do AME em crianças menores de 6 meses está abaixo das recomendações da OMS, situando-se em 45,8%^(6)^. Outro estudo nacional apontou que, apesar do aumento das taxas de AME entre 2015 e 2019, as taxas de AM misto também cresceram, especialmente em regiões com baixos índices de nutrição adequada^(7)^.

Somado a isso, no contexto atual, a mulher exerce diversos papeis na sociedade, sendo que a conciliação entre eles e a maternidade é desafiadora, o que torna o cotidiano da amamentação uma experiência complexa que demanda o envolvimento de profissionais de saúde, da família, da comunidade e de instituições. A adequação da mulher no papel de nutriz, aquela que amamenta, não é espontânea e natural, pois recebe influência de normas socioculturais e expectativas pessoais preconcebidas^(8)^. Portanto, este é um processo complexo que requer a ressignificação da identidade social da mulher e adaptação da rotina às necessidades do lactente^(8)^. Essas mudanças evocam desdobramentos psíquicos e sociais que podem passar despercebidos por quem deveria apoiar a mulher, mas que podem representar desafios e barreiras ao estabelecimento da amamentação.

A sobrecarga da maternidade em mulheres que cuidam dos filhos de maneira solitária tem repercussões psíquicas e físicas importantes^(9)^. O sentimento de exaustão e vulnerabilidade no pós-parto, a recuperação da cesariana, o isolamento e a falta de conhecimento sobre como cuidar do bebê minam a autoconfiança das mulheres^(9,10)^. Além disso, o surgimento de fatores como a percepção de falhar na amamentação, sentimento de culpa, falta de apoio social e tempo de licença-maternidade insuficiente é um desafio para a continuidade do AM^(9,10)^. Nesses casos, a ausência de apoio é um dos fatores que contribuem para o desmame precoce, enquanto o apoio social contribui significativamente para o estabelecimento do AME^(9-11)^.

Assim, o suporte social percebido, ou rede de apoio (RA), é um dos aspectos fundamentais para o sucesso da amamentação, podendo ser definido de diversas maneiras na literatura, tratando-se de um sistema constituído por diferentes indivíduos pertencentes à esfera social que fornecem apoio em diferentes âmbitos da vida: emocional, material, educacional, entre outros^(12)^. Em estudo conduzido no Reino Unido, identificaram-se três tipos de RAs que foram denominadas como “apoio extensivo”, para as mães que receberam apoio da família, amigos e profissionais, “apoio familiar”, para aquelas que eram apoiadas principalmente pela família, exceto por profissionais, e “baixo apoio”, para quem relatou menor apoio entre todos os potenciais apoiadores^(13)^. E as mulheres que amamentaram por mais de dois meses foram mais propensas a relatar amplo apoio^(13)^.

O AM é uma fase do ciclo gravídico-puerperal que não deve ser vivida de maneira solitária pelas nutrizes, pois, com suporte de RAs, há um alívio da sobrecarga da puérpera, o que permite que este período seja experienciado de maneira mais saudável e haja continuidade do AM^(14-16)^. Desse modo, objetiva-se conhecer a RA de mulheres que amamentam e seus impactos na amamentação. Entende-se que esse conhecimento pode ser um facilitador para o desenvolvimento de estratégias para promoção e proteção ao AM.

## OBJETIVOS

Conhecer a RA de nutrizes com filhos nascidos em dois hospitais de ensino no interior de São Paulo, sua efetivação no período puerperal e seus impactos na amamentação.

## MÉTODOS

### Aspectos éticos

O estudo atendeu a todas as recomendações da Resolução nº 466, de 12 de dezembro de 2012, do Conselho Nacional de Saúde, e foi aprovado pelo Comitê de Ética em Pesquisa da Universidade Estadual de Campinas (UNICAMP). Todas as participantes assinaram o Termo de Consentimento Livre e Esclarecido, e foram informadas de que poderiam retirar o consentimento a qualquer momento, mesmo após tal assinatura, sem sofrer qualquer tipo de ônus ou constrangimento.

### Tipo de estudo, local e participantes

Trata-se de estudo de coorte prospectivo, realizado em dois hospitais públicos de ensino, credenciados à Iniciativa Hospital Amigo da Criança, que atendem gestantes em seu ciclo gravídico-puerperal, sendo referência na região para o atendimento a gestantes de risco habitual, médio ou alto risco em processo de parto e nascimento. Aqui serão chamados de hospital A e hospital B. O tamanho amostral foi de 304 puérperas. Essa amostra foi dividida proporcionalmente entre os hospitais A e B: 156 e 148 participantes, respectivamente.

Tratou-se de amostra por conveniência e, dessa forma, foi composta exclusivamente por mulheres cisgênero. Por tanto, no decorrer do artigo, utilizaremos os termos “puérperas”, “mulheres” e “materno”.

### Critérios de inclusão

Incluíram-se mulheres com filhos em amamentação, que não precisaram em qualquer momento de observação em unidade neonatal de internação para cuidados intensivos ou intermediários, com idade igual ou maior a 18 anos, com ou sem comorbidades, desde que tais comorbidades não as impossibilitem de amamentar, com mais de 24 horas de pós-parto, amamentando filho de gestação única e fluência para comunicação na língua portuguesa do Brasil.

### Critérios de exclusão

Excluíram-se mulheres que apresentavam limitações para comunicação, bem como dificuldade para compreensão das perguntas, segundo diagnóstico médico e/ou percepção do pesquisador. Foram descontinuadas mulheres que foram separadas de seus filhos quando estavam em domicílio ou em casos em que houve óbito de um dos membros da díade (mãe ou lactente).

### Procedimento de coleta

Foram colhidos dados de prontuário, e realizada entrevista com questionário estruturado desenvolvido para o presente estudo. O questionário contemplou dados para caracterização da amostra, dados obstétricos e de amamentação, informações sobre o nascimento, bem como investigação sobre o reconhecimento pela puérpera quanto à RA.

A coleta de dados ocorreu em quatro tempos distintos. No primeiro tempo (T0), colheram-se dados junto às puérperas em alojamento conjunto, com 24 horas ou mais de pós-parto; no segundo, terceiro e quarto tempos, ocorreu coleta de dados junto a essas mesmas mulheres por telefone após um mês (T1), três (T2) e seis (T3) meses do nascimento do bebê. Como critério de continuidade das entrevistas, era necessário estar amamentando ou que o bebê estivesse recebendo leite de suas nutrizes de alguma forma. Assim, em T1, foram entrevistadas 272 puérperas (90,13% da amostra inicial), em T2, 224 (91,80% de T1), e em T3, tivemos 191 (96,46% de T2) entrevistas.

A coleta de dados em alojamento conjunto ocorreu de setembro de 2022 a maio de 2024, finalizando os contatos em T3 em novembro de 2024.

### Análise dos dados

Ocorreu análise estatística descritiva e inferencial aplicando o *software Statistical Package for the Social Sciences*. Para distribuição das variáveis sociodemográficas e clínicas, foi realizada análise descritiva por meio do cálculo de frequência e porcentagens, bem como medidas de tendência central e dispersão. As associações entre os grupos foram avaliadas por meio do teste qui-quadrado. Quando os pressupostos do teste qui-quadrado não foram atendidos, foi aplicado o teste de McNemar. O nível de significância adotado nas análises foi igual a 5%.

## RESULTADOS

Foram entrevistadas 304 mulheres, sendo 156 (51,32%) do hospital A e 148 (49,68%) do hospital B, conforme previsto no cálculo amostral. O perfil sociodemográfico das entrevistadas está apresentado na Tabela 1.

**Tabela 1:** Perfil sociodemográfico das entrevistadas em alojamento conjunto (N=304), Campinas, São Paulo, Brasil, 2023-2024

	n	%
**Faixa etária**		
18 a 34 anos	232	76,32
35 a 46 anos	72	23,68
**Cor da pele (autodeclarada)**		
Parda	155	50,99
Branca	117	38,49
Preta	31	10,20
Amarela	1	0,33
**Situação conjugal**		
Unida	265	87,17
Não unida	39	12,83
**Tempo de estudo (anos)**		
< cinco	9	2,96
Seis a nove	42	13,81
Dez a 13	227	74,67
> 14	26	8,55
**Renda familiar**		
Sem rendimento	2	0,66
Menos de um salário mínimo	26	8,55
De um a dois salários mínimos	112	36,84
De três a cinco salários mínimos	115	37,83
Mais de cinco salários mínimos	34	11,18
Não sabe/não quis informar	15	4,93

A média de idade das entrevistadas foi de 29,30 anos (mediana: 29; mín.: 18; máx.: 46; DP: 6,53). O tempo médio de estudo foi de 11,40 anos (mín.: 2; máx.: 22; DP: 2,37). Em consonância com as diretrizes do Sistema Único de Saúde (SUS), os municípios de residência prevalentes foram os municípios onde estão alocados os hospitais, seguidos de municípios vizinhos. Apenas nove (3%) mulheres residiam em áreas rurais.

Um pouco mais da metade (n=164; 53,95%) das entrevistadas afirmaram estar empregadas e, entre elas, 148 (80%) gozam do direito da licença maternidade. Sobre a situação conjugal, 265 (87,17%) das mulheres declararam estar unidas (sendo casadas, em união estável ou vivendo na mesma residência que o parceiro), com tempo de união médio de 6,54 anos (mín.: 3 meses; máx.: 27,16 anos; DP: 5,51 anos).

Sobre o histórico gestacional, o número médio de gestações foi de 2,51 (min.: 1; máx.: 9; DP: 1,52), e 106 (34,87%) eram primíparas. Apenas uma mulher (0,3%) não fez pré-natal. No entanto, 22 (7,26%) tiveram número de consultas inferior a seis, estando aquém do recomendado pelo Ministério da Saúde. A média do número de consultas de pré-natal foi de 10,53 (mín.: 2; máx.: 23; DP: 3,48). Quanto à via de parto, 157 (51,64%) tiveram seus filhos por via cirúrgica.

A respeito da maturidade fetal, 272 (89,47%) RNs apresentaram método de Capurro maior ou igual a 37 semanas (média: 38,66; mín.: 35; máx.: 41,43 semanas; DP: 1,35). O peso médio ao nascer foi de 3.122,77g (mín.: 2.004 g; máx.: 4.310 g; DP: 468,58 g).

Em relação ao histórico de AM, 239 (78,62%) puérperas afirmaram ter sido amamentadas. Havia 194 (63,82%) mulheres que já tinham outros filhos, sendo que 185 (95,36%) tiveram algum grau de experiência com a amamentação.

Quando questionado se as entrevistadas se sentiam apoiadas pela equipe do hospital para amamentar, verificou-se que 295 (97,04%) afirmaram que sim: aqui não foi indagada a categoria profissional, mas se de modo geral haviam recebido apoio. Entre as que reconheceram o apoio, 275 (90,46%) referiram recebê-lo sempre que precisavam, e 20 (6,58%) mencionaram que ele ocorreu algumas vezes ao longo do dia, não representando a totalidade de suas necessidades. Apenas nove (3%) não se sentiram apoiadas pela equipe. Apesar de a maioria se sentir apoiada para amamentar, 278 (90,46%) referiram não se sentirem seguras para amamentar com as informações que receberam no hospital.

Sobre o uso de fórmulas lácteas, para 76 (25%) dos RNs, havia prescrição de complementação. Porém, 87 (28,62%) puérperas afirmaram que seu filho havia recebido outro leite além do seu. A respeito do desejo materno sobre a continuidade da amamentação após alta hospitalar, apenas uma mulher referiu que não continuaria a amamentação, sendo a necessidade de trabalhar o motivo pela interrupção da lactação.

Sobre a presença de acompanhante, 280 (92,11%) mulheres o tiveram durante o trabalho de parto e parto. Apenas 77 (25,3%) amamentaram na sala de parto, e 120 (39,47%) referiram algum tipo de dificuldade para amamentar nas primeiras 24 horas de vida do bebê. Ter tido acompanhante durante o trabalho de parto e parto não impactou a possibilidade de amamentação ainda em sala de parto (p=0,9692), a oferta de fórmula durante a internação (p=0,9764) e nem a percepção materna sobre dificuldade para amamentar (p=0,8397). Não houve significância estatística na presença do acompanhante no processo de parturição na amamentação intra-hospitalar, conforme demonstrado na Tabela 2.

**Tabela 2:** Presença do acompanhante no trabalho de parto e parto e sua relação com a amamentação intra-hospitalar (N=304), Campinas, São Paulo, Brasil, 2023-2024

Variável	Acompanhante durante o trabalho de parto e parto				Valor de *p* ^*^
	Não		Sim		
	n	%	n	%	
**Amamentação na sala de parto**					0,9692^*^
Não	18	75,00	209	74,64	
Sim	6	25,00	71	25,36	
**Oferta de fórmula láctea durante a internação**					0,9764^*^
Não	17	70,83	197	71,12	
Sim	7	29,17	80	28,88	
**Dificuldade para amamentar**					0,8367^*^
Não	15	62,50	169	60,36	
Sim	9	37,50	111	39,64	

* *Valor de p obtido por meio do teste qui-quadrado.*

Quando questionadas sobre a RA que poderiam ajudá-las no período puerperal, 283 (93,09%) referiram que poderiam contar com ajuda após alta; dez (3,29%) não tinham certeza se teriam alguém que pudesse auxiliá-las; e 11 (3,62%) disseram não ter RA. O companheiro (marido, namorado, pai do bebê) foi o mais citado (n=177; 62,99%) dentro da RA, seguido da mãe (n=132; 46,98%), da sogra (n=40; 14,23%) e da irmã ou irmão (n=32; 11,39%). Duas (0,71%) mulheres não nomearam quem seria sua RA.

Como mencionado no método, após a primeira entrevista em alojamento conjunto, houve nova entrevista por telefone após um mês pós-parto (T1). Para as díades que continuavam em AM, foram feitos novos contatos após três (T2) e seis (T3) meses do nascimento. A amostra variou no decorrer das entrevistas, no primeiro contato telefônico, após um mês do nascimento do bebê (T1). Selecionou-se uma amostra de 272 puérperas (90,13% da amostra inicial); destas, 30 mulheres (11,02%) não amamentavam mais seus bebês. Em T2, foram entrevistadas 224 (92,56% da amostra anterior) puérperas, com 26 (11,61%) casos de desmame, e em T3, foram entrevistadas 191 (96,46% de T2) puérperas, com 19 (9,95%) casos de desmame. Ao longo da coleta, apenas duas (0,66%) puérperas foram descontinuadas. Não foi possível contato com 55 (18,09%) mulheres.

Em T1, as mulheres foram questionadas se tiveram RA durante o primeiro mês pós-parto, e foi estatisticamente significante o reconhecimento prévio de RA com sua efetivação no pós-parto (Tabela 3).

**Tabela 3:** Efetivação da rede de apoio em domicílio questionada em T1, Campinas, São Paulo, Brasil, 2023-2024

Crença de que terá RA em casa (Questionado em T0)	Presença de RA no primeiro mês pós-parto		Valor de *p* ^*^
	Não	Sim	
Não	4	6	**< 0,0001**
Sim	33	219	

* *Valor de p obtido por meio do teste de McNemar; RA - rede de apoio; T0 - momento em que a entrevista foi realizada: alojamento conjunto.*

Ao avaliar o impacto da presença de RA no uso de chupeta no primeiro mês de vida do bebê, não houve significância estatística (p=0,6604), assim como a introdução de fórmula ao longo dos 6 meses de vida do bebê, questionada em T1 (p=0,3025), T2 (p=0,3746) e T3 (p=0,0709).

Porém, verificou-se relação positiva entre as mulheres que tinham RA e possibilidade de descanso ao longo do dia, mostrando favorecer o AME ao sexto mês do bebê (p=0,0424). Ter RA não interferiu no descanso do período noturno (Tabela 4). Entre as mulheres que afirmaram estar alimentando seus bebês exclusivamente com leite materno no sexto mês de vida (n=76), 15 delas (19,74%) ofertaram fórmula láctea em algum momento nos meses anteriores. Assim, entre todas as entrevistadas, apenas 20,07% (n=61) tiveram AME até o sexto mês e, para isso, a RA apresentou significância estatística, como mostra a Tabela 4.

**Tabela 4:** Rede de apoio no primeiro mês pós-parto e associação com aleitamento materno, Campinas, São Paulo, Brasil, 2023-2024

Variável	RA no primeiro mês pós-parto				Valor de *p* ^*^
	Não		Sim		
	n	%	n	%	
**Descanso ao longo do dia (T1)**					**0,0005**
Não	22	56,41	66	28,33	
Sim	17	43,59	167	71,67	
**Descanso à noite (T1)**					0,5510
Não	16	41,03	84	36,05	
Sim	23	58,97	149	63,95	
**Alimentação do bebê aos 6 meses**					**0,0424**
Outros^#^	19	79,17	96	57,49	
AME	5	20,83	71	42,51	
**AME até o sexto mês**					**0,0490**
Não	35	89,74	176	75,54	
Sim	4	10,26	57	24,46	

* *Valor de p obtido por meio do teste qui-quadrado; RA - rede de apoio; AME - aleitamento materno exclusivo; T1 - um mês pós-parto; #Outros: aleitamento materno parcial, misto ou complementado.*

## DISCUSSÃO

Os dados sociodemográficos se assemelham aos achados de pesquisas realizadas pela Rede Cegonha e Nascer Saudável^(14)^ em hospitais públicos e conveniados ao SUS pelo Brasil, os quais apresentam uma maior porcentagem de mulheres com idade inferior a 35 anos e pardas. Entretanto, houve divergência entre os dados referentes ao tempo de escolaridade encontrada no setor público, pois, na pesquisa da Rede Cegonha, foi de dez anos de estudo. Nessa amostra, 100% atendida pelo SUS, assemelhou-se à escolaridade encontrada no setor privado, que foi majoritariamente de 11 a 13 anos^(14)^.

A maioria das mulheres entrevistadas (97,04%) se sentiu apoiada pela equipe do hospital, sendo esse um fator protetivo ao enfrentamento de dificuldades na amamentação, como mostra estudo que revelou que mulheres que perceberam apoio adequado à amamentação ainda no hospital tiveram menores chances de enfrentar dificuldades com a amamentação, em comparação com aquelas que consideraram esse apoio inadequado^(15)^.

Apesar de se sentirem apoiadas pela equipe, referiram não sentir segurança para amamentar a partir das informações recebidas, o que denota a importância do fortalecimento da educação em saúde e do acompanhamento pós-alta. A literatura descreve que a falta de confiança no conhecimento adquirido sobre a amamentação pode indicar um apoio educacional insuficiente. Essa demanda deve ser atendida, pois mulheres que têm conhecimento suficiente sobre a amamentação têm maiores chances de amamentar por mais tempo^(16)^.

Estudo do tipo quase-experimental, conduzido com puérperas no pós-parto imediato, demonstrou como o apoio planejado e de longo prazo oferecido por enfermeiras se relaciona com pontuações mais altas na escala de autoeficácia na amamentação, que consiste no quanto a mulher acredita em sua capacidade para amamentar. Neste estudo, o apoio das enfermeiras, por meio do compartilhamento de informações e de uma comunicação eficaz, contribuiu significativamente para o aumento da autoeficácia materna, fator que influencia o sucesso e a continuidade da amamentação^(17)^.

No presente estudo, a maioria das participantes (93,09%) afirmou poder contar com RA após a alta. Sabe-se da importância da presença da família antes mesmo do nascimento do bebê, em especial do acompanhante da mulher durante todo o processo de parturição^(18)^. O apontamento do companheiro como principal fonte de apoio deve ser visto como algo relevante para continuidade da amamentação, uma vez que o apoio do pai da criança por mais de 24 meses é considerado um fator de proteção ao AM^(16)^.

A RA reconhecida no alojamento conjunto foi confirmada no primeiro mês pós-parto, sendo estatisticamente significante (p=0,0001). Ou seja, 92,64% das mulheres entrevistadas, após um mês pós-parto, validaram a participação da RA. Ao encorajar os companheiros e companheiras das nutrizes a reconhecer o papel crucial que desempenham na nutrição dos filhos, desde o início da amamentação, estimula-se a sua participação ativa nos cuidados diários da criança e em outras atividades, que podem contribuir para uma experiência de amamentação mais positiva para as mães^(19)^. Os profissionais de saúde podem atuar no reconhecimento e fortalecimento desse papel desde o acompanhamento pré-natal.

Além dos aspectos emocionais e sociais, sabe-se que a amamentação pode ser um período de grande demanda fisiológica para a mãe, já que ela deve fornecer os nutrientes necessários para a criança através do leite, o que implica que ela requer cuidados de ordem física e emocional, além de repouso. O ciclo vigília/sono pode ser drasticamente alterado pela amamentação, assim como pode ser mais estimulada a secreção de hormônios e neurotransmissores envolvidos na “reação de luta e fuga”^(20)^. Estudos sugerem que a maior secreção de prolactina e ocitocina diminui a resposta ao estresse materno, ansiedade e irritabilidade^(21)^. Além dos efeitos hormonais, a RA pode ser mais um fator para diminuir a resposta ao estresse. O presente estudo mostrou que a RA favorece a possibilidade de descanso no período diurno (p=0,0005). Porém, não apresentou impacto sobre o descanso no período noturno (p=0,5510), o que pode ser decorrente de menor disponibilidade da RA nesse período.

A correlação do uso de chupeta com a presença da RA não foi estatisticamente significativa (p=0,6604). Uma possível explicação pode ser a falta de conhecimento sobre recomendações quanto ao uso da chupeta, como analisado em recente estudo que investigou o conhecimento e as atitudes dos pais em relação à amamentação^(22)^. Neste estudo, os pais demonstraram conhecimento a respeito dos benefícios do AM para a díade, do contato pele a pele, da prática de alojamento conjunto, da amamentação sob livre demanda e da importância de seu envolvimento na alimentação do bebê, porém demonstraram falta de conhecimento sobre os impactos do uso da chupeta^(22)^.

Apesar de os estudos apontarem que a presença de acompanhante em sala de parto e da RA no período puerperal contribui para melhores taxas de amamentação^(23)^, a RA não impactou a introdução de fórmula láctea antes ou após alta. Também não houve significância estatística entre ter acompanhante durante o trabalho de parto e parto, e a amamentação na sala de parto, oferta de fórmula láctea intra-hospitalar, bem como dificuldade para amamentar. Há algumas barreiras para a continuidade da AM, como a existência de uma forte narrativa generalizada de que os corpos das mulheres são inadequados e que elas não conseguem produzir volume suficiente e/ou leite de qualidade. Também é veiculado que a alimentação com fórmula é mais conveniente e socialmente aceitável, o que, obviamente, é apoiado por empresas de fórmulas lácteas para vender mais seus produtos^(4)^.

Vencidas as dificuldades existentes nos primeiros meses da amamentação, o presente estudo mostrou uma relação positiva entre a presença da RA, a retirada da fórmula láctea e a retomada do AME até o sexto mês, e do AME do nascimento aos 6 meses. Esse achado corrobora estudo que diz que a percepção de um maior nível de apoio social está relacionada a um maior tempo de amamentação, ou seja, mulheres que percebem que têm maior apoio têm maiores chances de planejar e realizar um AM prolongado^(20)^. Evidências sugerem que o apoio social melhora os resultados da amamentação^(13)^.

A continuidade do AME após a alta hospitalar está relacionada ao enfrentamento pelas nutrizes de dificuldades relacionadas à amamentação. Entre tais dificuldades, podem surgir a percepção de produção insuficiente de leite, limitações para encontrar boas posições para amamentar, dificuldades do bebê em pega e sucção, introdução de suplementação por fórmulas lácteas, saberes inadequados sobre o manejo para iniciar ou desmamar da suplementação, assim como desconfortos e dor durante a amamentação^(8,24)^. Além disso, o apoio insuficiente para o enfrentamento das dificuldades pode ser um fator contribuinte para o desmame precoce. A sobrecarga gerada pela necessidade de conciliação entre o papel de nutriz e outras atividades diárias acarreta a impossibilidade de descanso relatado pelas puérperas^(8,9)^. Logo, a RA se mostra fundamental para o enfrentamento dessas dificuldades, cuidados e fortalecimento da nutriz, além de proteção e promoção do AM.

Com base na literatura, os serviços de saúde pública e as intervenções que visam melhorar as taxas de amamentação devem ter como alvo principal o apoio informativo, social e emocional às mães, refletindo uma abordagem individual de mudança de comportamento^(13)^. Fortalecer esses vínculos desde o pré-natal, ajudar a gestante a reconhecer sua RA e identificar casos de apoio insuficiente são cruciais para elaboração de um plano de cuidados individualizado, preenchendo as lacunas que se mostram durante as consultas. Por fim, com RA estruturada, a nutriz terá maior probabilidade de aleitar, recebendo apoio, proteção e promoção do AME e AM prolongado.

### Limitações do estudo

A limitação deste estudo reside na impossibilidade de descrever quais são as ações que as mulheres reconhecem como apoio ao longo do pós-parto que, a partir da ótica delas, mais impactaram diretamente as relações que permeiam a amamentação. Assim, o delineamento do presente estudo não se deu de forma a favorecer a análise das ações vivenciadas pelas mulheres ao longo do tempo que representam o apoio e proteção da amamentação que elas esperam. Estudo qualitativo poderá responder aspectos relacionados ao significado de RA para as puérperas.

### Contribuições para as áreas da enfermagem e saúde pública

Espera-se que este estudo contribua para destacar a relevância da RA à amamentação, propiciando subsídio para ações políticas e assistenciais, bem como futuras investigações científicas sobre este tema e com a consequente promoção e apoio ao AM. Os impactos positivos da RA devem ser discutidos e promovidos no ensino, na pesquisa e prática clínica, por se tratar de tecnologia leve do cuidado possível de ser implantada na prática diária do cuidado à mulher no ciclo gravídico-puerperal.

## CONCLUSÕES

A maioria das puérperas reconhece a equipe de saúde do hospital como RA. Consideram que, após a alta, o companheiro (marido, namorado, pai do bebê) será uma provável fonte de apoio, seguido do apoio da mãe, da sogra e da irmã ou irmão. Após um mês pós-parto, quando as mulheres foram entrevistadas, verificou-se que a RA familiar se efetivou, com as mulheres validando essa experiência. A presença de RA favoreceu o descanso no período diurno e aumentou a probabilidade de AME no sexto mês.

Desse modo, o reconhecimento da RA pelas nutrizes poderá ser fator facilitador para superação de desafios, promoção do AM e maiores chances de um AM prolongado. Nesse contexto, a equipe de saúde deve atuar de forma intencional e ativa, desde o pré-natal, incluindo a família, bem como auxiliando a mulher a identificar e fortalecer sua RA, a fim de auxiliá-la no estabelecimento e continuidade da amamentação.

## Data Availability

Os dados de pesquisa estão disponíveis em repositório: https://doi.org/10.25824/redu/IJFTL1.
